# Allosteric interactions among voltage-sensor modules of sodium channels probed by scorpion toxin modifiers

**DOI:** 10.46439/neurobiology.4.021

**Published:** 2022

**Authors:** Michael Gurevitz, Boris S. Zhorov, Ke Dong

**Affiliations:** 1 Department of Plant Molecular Biology & Ecology, George S. Wise Faculty of Life Sciences, Tel-Aviv University, Ramat Aviv, Tel Aviv 69978, Israel; 2 Department of Biochemistry and Biomedical Sciences, McMaster University, Hamilton, L8S 4K1 Canada; 3 Sechenov Institute of Evolutionary Physiology & Biochemistry, Russian Academy of Sciences, St. Petersburg, 194223, Russia; 4 Department of Biology, Duke University, Durham, NC, USA

## Abstract

Gating of voltage-dependent sodium channels involves coordinated movements of the voltage sensors in the voltage-sensing modules (VSMs) of the four domains (DI-DIV) in response to membrane depolarization. Zhu et al. have recently examined the effects of charge reversal substitutions at the VSM of domain III on the action of scorpion alpha- and beta-toxins that intercept the voltage sensors in domains IV and II, respectively. The increased activity of both toxin types on the mutant channels has suggested that the VSM module at domain III interacts allosterically with the VSM modules in domains IV and II during channel gating thus affecting indirectly the action of both scorpion toxin classes.

## Probing Sodium Channel Dynamics with Scorpion Toxins

Voltage-gated sodium channels (Navs) are membrane proteins composed of alpha and beta subunits. The ~260 kDa pore-forming **α**-subunit is organized in four repeat domains (DI-DIV). Each domain contains six transmembrane **α**-helical segments (S1-S6) and a membrane re-entrant P-loop (SS1-SS2) between S5 and S6 (Pore region), all connected by intra- and extra-cellular loops. The S4 segment of each domain contains 4–8 positively-charged residues at three-residue intervals and functions as a voltage-sensor [1,2]. Upon membrane depolarization, the sensors move in the extracellular direction, thereby rendering channel activation and pore opening. Channel inactivation is mediated by the IFM (isoleucine-phenyl alanine-methionine) motif in the short intracellular loop connecting DIII and DIV [2,3].

Due to their critical function in excitability, Navs are targeted by a large variety of toxins used by venomous animals for prey and defense. Scorpion toxins that affect these channels are divided between two classes [4,5]: **α**-toxins that bind at the pharmacologically-defined site-3, inhibiting channel inactivation by preventing the outward movement of the voltage sensor in domain IV (DIV-S4) [6,7], and **β**-toxins that bind at the pharmacologically-defined site-4, enhancing channel activation by trapping the voltage sensor of domain II (DII-S4) in its outward position [8–12]. Each class is further divided to distinct pharmacological groups based on their binding features [13–15].

Systematic mutagenesis at extracellular loops that connect the trans-membrane segments, illuminated channel regions involved in toxin selectivity toward mammalian versus insect Navs, as well as amino acid residues involved in channel sensitivity to the toxins [6,8,11,12,16–20]. The large collection of toxin and channel mutants enabled double-mutant cycle analyses and association/dissociation assays, which raised putative pairwise interactions between toxin and channel amino acid residues. Using the anti-mammalian toxin Lqh2 (from the scorpion *Leiurus quinquestriatus hebraeus*) as a model of the alpha class, residues of the toxin Core-domain have been suggested to interact with channel residues at the voltage-sensing module of DIV in the rat brain channel Nav1.2a, thus providing a partial view of receptor site 3 [6,12,20,21]. Since the movements of S4 voltage-sensor at DIV have been implicated in the inactivation process of the channel [7,22], this mutational analysis substantiated at the molecular level the specific effect of scorpion alpha toxins on channel inactivation. Similar analyses using Css4 (from the scorpion *Centruroides suffusus suffusus*) as a representative of the beta class have suggested putative pairwise interactions of amino acids at the toxin core and the voltage-sensing module at DII of Nav1.2a [11,12,19], rationalizing the specific effect of scorpion beta toxins on channel activation. Both studies raised the possibility of toxin interactions also with the Pore-module of the channel, although the supporting experimental evidence was less definitive [19,20].

Considering that channel gating in response to membrane depolarization involves coordinated movements outwards and backwards of the four voltage sensors [2,23–26], the question that arises is how do the toxins bind to such mobile receptor sites, and what is the mechanism by which they convey their effects. Although these issues were extensively studied [9,10], they still remained unclear.

**Figure 1:**
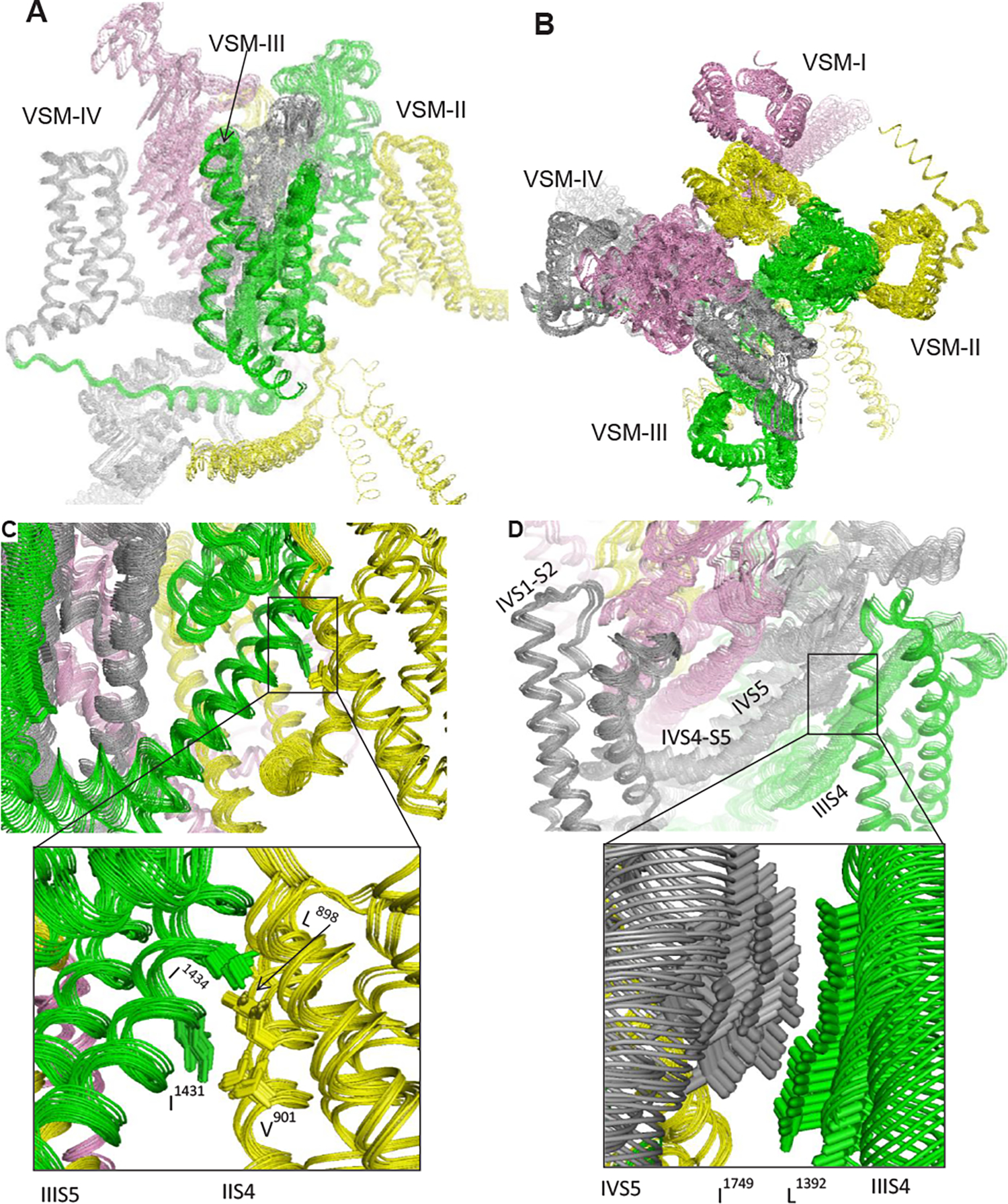
Motion transmission upon *in-silico* deactivation of voltage-sensing helix DIII-S4 in the cockroach sodium channel BgNav1–1a. In the intermembrane (**A**) and extracellular (**B**) views, repeat domains I, II, III and IV are in pink, yellow, green and gray, respectively. The channel model with activated VSMs and presumably inactivated Pore module was computed at the Compute Canada (www.computecanada.ca) using the AlphaFold2 software [34]. Then the Pore module was *in-silico* opened towards the cryo-EM structure of the open rNav1.5 channel [35] and helix DIII-S4 was stepwise shifted in the cytoplasmic direction using described methods [29,36]. Twenty-one snapshots were superimposed with PyMol (Version 0.99rc6; Schrödinger, LLC, New York, NY). The forced downshift of helix DIII-S4 caused perturbations all over the channel. In particular, the shift was transmitted to the linker-helix DIII-S4-S5 and then to DII-VSM through hydrophobic contacts between DIII-S5 and DII-S4 (**C**). Helix DIV-S5 shifted down due to a hydrophobic contact with DIII-S4 (**D**), and through linker-helix DIV-S4-S5 the motion was transmitted to DIV-VSM.

In a combined experimental approach that included site-directed mutagenesis of toxins and channels expressed heterologously [12], accompanied by binding studies, electrophysiological functional assessments, and three-dimensional modeling, receptor sites 3 and 4 were recently described in an insect voltage-gated sodium channel [27–29]. In these studies, Song *et al.* [27] examined whether the VSM at DIII was involved in the action of a scorpion depressant **β**-toxin, Lqh-dprIT3 [30] on a cockroach channel splice variant, BgNav1–1 [31]. To their surprise they discovered that this channel variant was hypersensitive to the toxin due to an L1285P substitution in DIII-S1 resulting from a U-to-C RNA-editing event. Furthermore, they found that charge-reversal of a negatively-charged residue (E1290K) at the extracellular end of DIII-S1 and the two innermost positively-charged residues (R4E and R5E) in DIII-S4 also increased the channel sensitivity to Lqh-dprIT3 raising questions as to the mechanism involved. Further analysis of substitutions R4E and R5E in DIII-S4 has also shown increase in activity of two site- 3 toxins, Lqh**α**IT from the scorpion *Leiurus quinquestriatus hebraeus* and Av3, an insect- selective toxin from the sea anemone *Anemonia viridis* [28]. Moreover, charge- reversal of either of two conserved negatively-charged residues, D1K and E2K, in DIII-S2 increased the action of both site-3 as well as site-4 toxins. Homology modeling has suggested that S2-D1 and S2-E2 interact with S4-R4 and S4-R5, respectively, in DIII-VSM, in the activated state of the channel [28]. On the basis of these results the authors have suggested that the effects of charge-reversal of ionizable residues in DIII-VSM are allosterically transduced to the extracellular linkers of the VSMs of domains II and IV, leading to prolongation of toxin binding at sites-3 and 4, thus increasing their effects. These results have shown that the movement of the voltage-sensor at domain III is linked to the movements of the voltage-sensors at domains IV and II, thus demonstrating the dynamics of the channel during gating and coordinated movement of all four voltage-sensors in the four channel domains. Possible pathways of motion transmission from DIII-VSM to other modules of the channels are presented in Figure 1.

Despite these thought-provoking results the mechanism by which the toxins sustain their grip over the channel upon membrane depolarization and outward movement of the voltage-sensors is still unclear. In assuming that toxin binding is a stepwise process that follows the principles of the ‘Induced fit theory’ [32], where binding begins with recognition of complementary shapes and continues with molding of amino acid side-chains that strengthen the interaction, the arising question is what happens to the complex upon membrane depolarization, when the S4 segments move toward their outward activated state. It is possible that the toxin accommodates to the conformational alterations by interaction with a different subset of channel residues at the voltage-sensing module sustaining its hold over the channel and does not fall-off due to its interaction with the less labile Pore-module [19,33]. A partial example to such a scenario has recently been described by structural modeling of the interaction of Lqh-dprIT3 with the cockroach sodium channel [29]. The modeling accompanied with mutational analysis has suggested that the toxin approaches the salt-bridge between R1 and D802 at DII-VSM to form contacts with linkers DII-S1-S2, DII-S3-S4, DIII-P5- P1 and DIII-P2-S6. Elimination of this salt-bridge enables deeper penetration of the toxin into a cleft at DII-VSM to form new contacts with the channel, leading to increased channel sensitivity to the toxin. Strong depolarizations might detach the toxin from its binding site, a scenario that not necessarily occurs under weak to moderate physiological changes in membrane potential. In any event, the toxin-channel interaction involves transient conformational intermediates of the channel, and therefore it seems at present that a comprehensive clarification of the way scorpion toxins interact with Navs is more challenging than anticipated and likely requires determination of the structures of the channel-toxin intermediary complexes. The structural models constructed by Zhorov *et* al [29] are leading steps in this direction.
